# Syringomyelia Is Associated with a Reduction in Spinal Canal Compliance, Venous Outflow Dilatation and Glymphatic Fluid Obstruction

**DOI:** 10.3390/jcm12206646

**Published:** 2023-10-20

**Authors:** Grant Alexander Bateman, Alexander Robert Bateman

**Affiliations:** 1 Department of Medical Imaging, John Hunter Hospital, Newcastle, NSW 2305, Australia; 2 Faculty of Health, Callaghan Campus, University of Newcastle, Newcastle, NSW 2308, Australia; 3 School of Mechanical Engineering, University of New South Wales, Sydney, NSW 2502, Australia; alexander.bateman@unsw.edu.au

**Keywords:** syringomyelia, compliance, glymphatic drainage, venous dilatation, impedance pump

## Abstract

The cause of the cystic dilatation of the cord found in syringomyelia has been a source of conjecture for a considerable time. Recent studies have shown that there is a reduction in craniospinal compliance in both childhood hydrocephalus and multiple sclerosis which leads to venous outflow dilatation. Both diseases are associated with glymphatic outflow obstruction. Venous dilatation will narrow the perivenous glymphatic outflow pathway and lead to an increase in glymphatic outflow resistance. Syringomyelia has been shown to be associated with reduced spinal canal compliance. This paper discusses the possibility that venous dilatation and obstructed glymphatic outflow within the cord may be behind the cystic dilatation found within syringomyelia.

## 1. Introduction

**Figure 1:**
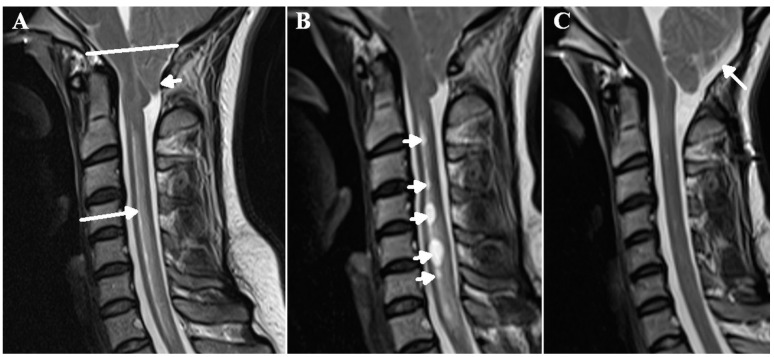
The progression of edema in syringomyelia. A T2 weighted sagittal MRI of the neck in a 28-year-old female patient who presented with headache on bending and coughing. There is a Chiari 1 malformation with significant cerebellar tonsil herniation (small white arrow) below the foramen magnum (white line) leading to reduced spinal compliance. There is spinal cord edema from C2 to T1 (long arrow) (**A**). One year later, following conservative management, there were multiple cystic cavities developing (arrows) (**B**). Following posterior fossa decompression (arrow), there is now improved compliance in the spinal canal with the resolution of the edema and the cystic cavities previously noted (**C**).

Syringomyelia is the development of a tubular, fluid-filled cavity within the parenchyma of the spinal cord. It is commonly associated with an intradural, extramedullary obstruction such as the tonsillar herniation found in Chiari 1 malformation, Dandy-Walker malformations, arachnoiditis secondary to meningitis, trauma or hemorrhage and compressive tumors such as meningioma [1]. Intramedullary causes such as direct spinal cord trauma and intrinsic tumor are also noted [2]. The cause of the cyst formation has been a source of considerable debate over the years. Most hydrodynamic theories invoke mechanisms whereby cerebrospinal fluid (CSF) from the subarachnoid space is pumped into the spinal cord parenchyma. Elliott et al. reviewed these theories and suggested they were all wanting [3]. One of the current authors developed a theory that syringomyelia was directly related to a reduction in spinal canal compliance and increased pulsation strength analogous to those in normal-pressure hydrocephalus (NPH) [4]. It was noted that both disorders are associated with the accumulation of fluid within the center of their respective neural structures, with the displacement of the parenchyma into the subarachnoid space, despite the lack of a well-defined pressure gradient [4]. It was envisaged that an alteration in impedance matching between the venous outflow of the brain or spinal cord and the extradural outflow pathways would increase venous pressure [4]. However, there was no available hydraulic mechanism to account for this suggestion. Later, the hypothesis was further developed to include the disruption of the blood–spinal cord barrier. An increase in pulse pressure within the veins draining the cord would lead to capillary disruption and an increase in interstitial fluid production [5]. Indeed, in syringomyelia associated with a Chiari 1 malformation, cord edema develops before syrinx formation occurs [6] (See Figure 1A,B). However, the accumulation of this interstitial fluid would require an obstruction of the outflow pathway, which was unaccounted for in the original theory. Recently, the dilatation of the superficial cortical veins draining the brain has been noted in children with hydrocephalus [7]. Modelling suggested that dilatation could only be brought about via an increase in the venous transmural pressure of approximately 4 mmHg [7]. It was suggested that a change in impedance pumping could account for this finding [7]. It has now been recognised that this same phenomenon could produce an increase in venous pressure within the cord in syringomyelia. The second component of the theory, i.e., the obstruction of the outflow of interstitial fluid, could also be explained by venous dilatation. It is the purpose of the current paper to describe the interaction between spinal canal compliance, venous dilatation and interstitial fluid outflow obstruction in syringomyelia.

## 2. Discussion

### 2.1. Compliance and Venous Pressure in Hydrocephalus, Multiple Sclerosis and Syringomyelia

There is a correlation between hydrocephalus, multiple sclerosis and syringomyelia. In one paper looking at children with Chiari 1 malformation, 58% were found to have syringomyelia and 11% were found to have hydrocephalus [8]. Syringomyelia is also noted in 4.5% of patients with MS [9]. It is suggested that compliance changes may link all three diseases. Compliance is a measure of the change in pressure within a structure brought about by a change in volume [10]. In NPH, there is a significant reduction in craniospinal compliance [11]. Studies of the waveforms and flow volumes passing through the venous outflow in NPH indicate that cortical vein flow shows a high-impedance waveform before shunt insertion and a low-impedance waveform after shunt insertion [12]. Impedance is a measure of the ratio of the pressure to the flow through a system, taking into account both the pulsatile and non-pulsatile components [13]. In the absence of a pulsatile flow, impedance is equivalent to hydraulic resistance. The high-impedance waveform in NPH is flatter and clips the pulsatile flow, having the effect of reducing the venous flow volume [4]. The finding of a decrease in the blood flow volume through a venous territory despite the arterial inflow being unchanged is an indication of an increase in venous outflow pressure because as venous pressure rises, more of the inflow is diverted into collateral pathways to mitigate the blockage. Hydrocephalus in children is associated with a reduction in the venous return through the sagittal sinus equivalent to 12.9% of that of the arterial inflow [7], indicating an increase in venous pressure. This pressure increase has been found to be localised to the cortical veins, which were dilated by 22% in cross-section in this disease compared to that in controls, indicating a transmural pressure gradient increase of 4 mmHg [7]. It was hypothesised that the increase in this pressure was brought about by the hydraulic phenomenon called impedance pumping [7]. Hickerson et al. defines an impedance pump as a device which uses a mismatch in impedance between two adjacent tubes to drive fluid flow without valves [14]. The physiology of the venous outflow of the brain conforms to the four criteria Hickerson et al. have suggested to indicate if an impedance pump is operating [7]. Normally, the rhythmic compression of the cortical veins is hypothesised to aid venous outflow via this mechanism. Thus, either a loss of this pumping or a reversal of venous impedance pumping could account for the elevated venous pressures found in hydrocephalus.

The findings from patients with multiple sclerosis (MS) appear to be similar to those found for patients with hydrocephalus. The cortical veins in MS are 29% larger in cross-section than those in matched controls, suggesting an increase in venous transmural pressure of approximately 6.5 mmHg [15]. In syringomyelia secondary to Chiari 1 malformation, Capel et al. measured the arterial inflow and pulsation stroke volume at the skull base and compared it to the venous outflow from the sagittal and straight sinuses in patients with Chiari 1 malformation with and without syrinx formation [16]. They looked at the percentage of the arterial blood flow returning via the main venous sinuses and acknowledged that a reduced percentage suggested an increase in the blood returning via collateral pathways. They found that a decrease in the mobile compliance afforded via cervical CSF pulsation in patients with syrinxes was significantly associated with an increase in venous collateral flow, i.e., increased venous pressure. The finding of reduced compliance in syringomyelia is not new. Total intracranial compliance was reduced by 20% in one study [17] and by 28% in another in patients with Chiari 1 malformation [18] (see Figure 1A). Successful posterior fossa decompression surgery was associated with a 54%increase in this compliance [18]. In one study, posterior fossa decompression to increase compliance led to a resolution of the syrinx in 81% and a significant improvement in 19% of patients [19] (see Figure 1C). However, local spinal canal compliance is even more severely reduced than total craniospinal compliance in this disorder. In Chiari 1 malformation with syrinx formation, local cervical compliance is reduced by 45% with a 44% increase in CSF pulse pressure [20]. There is a reduction in compliance surrounding the cord in all forms of syringomyelia, not just in those secondary to foramen magnum obstruction, including those secondary to trauma and arachnoid scarring [20]. The cortical veins are too small to directly visualise in humans with modern imaging but in a kaolin-induced dog model of syringomyelia, microangiograms showed evidence of venous engorgement [21]. Laser Doppler flowmetry has shown a significant increase in regional spinal cord blood flow after the decompression of a syrinx [22], suggesting a reduction in blood flow resistance.

### 2.2. Dilated Outflow Veins and the Effect on Glymphatic Flow

**Figure 2:**
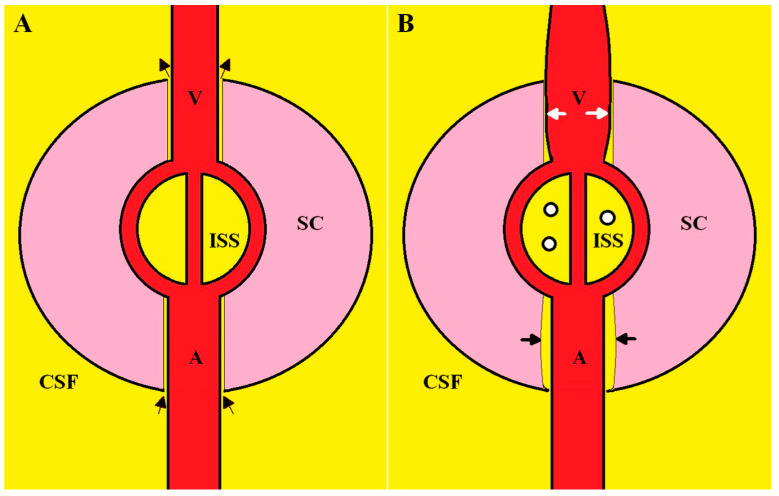
A diagrammatic representation of syringomyelia. Panel (**A**) is the normal physiology. SC is the spinal cord, CSF is the surrounding cerebrospinal fluid, A is the arteriolar inflow, V is the vein draining the spinal cord and ISS is the interstitial space. CSF enters the space between the artery and spinal cord via the arterial perivascular space (lower black arrows), passes through the interstitial space and exits via the venous perivascular space (upper black arrows). Panel (**B**) shows the changes producing syringomyelia. There is a dilatation of the vein compressing its perivenular space (white arrows). There is buildup of interstitial fluid increasing the ISS pressure and there is coalescence of small cystic spaces indicating the developing syrinx (small circles). The back-up of interstitial fluid dilates the arteriolar perivascular space (black arrows).

The glymphatic system is thought to represent a continuous movement of fluid from the subarachnoid space through the brain or spinal cord parenchyma and back into the subarachnoid space. In the glymphatic system, CSF enters the periarterial vascular space (which is limited by the pia mater) via arterial pulse-induced convection. From here, it enters the interstitial space through gaps in the astrocyte end-foot processes and via aquaporin 4 water channels. Water exits the interstitial space via the perivenous space [23] (see Figure 2A). The perivenous outflow from the brain has been visualised in humans following the opening of the blood–brain barrier using focused ultrasound [24]. However, some authors maintain that glymphatic flow within the cord, especially via the efflux route, remains highly debatable [11]. However, there is in existence long-standing evidence for this flow. In 1952, Bering used deuterium oxide (heavy water) as a water tracer, injected it intravenously in both a dog model and in healthy human subjects and determined the half-life of the exchange of this water to reach equilibrium between the capillary bed and the brain interstitial space in dogs and between the capillaries and CSF in humans [25]. In dogs, the half-life of the exchange of water between the capillaries and brain interstitial space was 12 s in grey matter and 20 s in white matter, indicating an almost instantaneous equilibration between the blood and interstitial space of the neuraxis [25]. In humans the half-life of the tracer was estimated within the subarachnoid space around the brain, the ventricles and the spinal canal via repeated CSF sampling. Using a single-compartment model, the half-life of the exchange and the known volume of distribution (the CSF volume), one of the current authors was able to estimate the turnover of water between the total neuraxis capillaries and the CSF space to be approximately 22 mL/min [26]. This figure is about two orders of magnitude higher than the net production of CSF from the choroid plexus and neuraxis combined, which is 0.35 mL/min [26]. This can only mean that water flows from the neuraxis interstitial space into the subarachnoid space and back into the interstitial space or else the net CSF production would be much higher. Another interesting finding is the relative glymphatic flow between the brain and spinal cord. The half-life between the blood and spinal canal CSF was 23.2 min. The spinal canal CSF space averages at 80 mL, giving a turnover rate of 2.4 mL/min in the spinal canal [26]. The volume of the spinal cord averages at 28 cm^3^ in humans [27], meaning the glymphatic flow of the spinal cord averages at 86 µL/min/cm^3^. The brain’s turnover rate is approximately 19.4 mL/min and brain volume averages at 1400 cm^3^ [28], giving a glymphatic flow averaging at 14 µL/min/cm^3^, suggesting that the normalised glymphatic flow is six times higher in the spinal cord than that in the brain parenchyma.

As the outflow pathway of the glymphatic system is thought to lie between the small veins and their pial covering, an increased intracranial venous volume would be expected to reduce the functional patency of the perivenous spaces [29]. A narrowing of the perivenous glymphatic outflow space would be expected to increase the outflow resistance of glymphatic fluid and lead to a reduction in the fluid flow [29]. This concept has been used to explain the diurnal variation in glymphatic flow, which is known to be elevated at night and low in the day in humans. Low intracranial pressure whilst erect reduces CSF pressure compared to venous pressure and dilates the cortical veins which are hypothesised to reduce glymphatic flow in the day compared to those in the supine position at night whilst sleeping [29]. As already discussed, there is evidence of dilated cortical veins in hydrocephalus and MS. There is also evidence of reduced glymphatic flow. In MS, the diffusion along the perivascular space index (DTI-ALPS) is a proxy for glymphatic function and was reduced compared to that in controls. The index was lower in progressive MS patients vs. relapsing remitting MS, associated with clinical disability and disease duration, lesion load and atrophy of grey matter [30]. In NPH, there is a similar reduction in the DTI-ALPS compared to that in controls [31]. The size of dilated perivascular spaces visible on MRI are also thought to reflect impaired glymphatic exchange [32]. A meta-analysis of nine papers suggested that enlarged perivascular spaces were increased in prevalence, number and volume in MS compared to those in controls [33]. Similarly, many animal models of syringomyelia have shown dilated perivascular spaces. In Cavalier King Charles spaniels with spontaneous syringomyelia, there are enlarged perivascular spaces filled with eosinophilic material [34]. In a rabbit model of syringomyelia initiated via kaolin-induced arachnoiditis, the perivascular spaces were expanded on microscopy [35]. A cat model of subarachnoid space scarring caused a significant dilatation of the perivascular spaces in central grey matter and white matter. The intramedullary pressure was consistently higher than the adjacent subarachnoid space pressure [36]. In a rat model of traumatic syringomyelia, arteriolar perivascular spaces were enlarged in the perisyrinx region but not above or below it [11]. In another rat model, alternating negative and positive intrathoracic pressures from spontaneous breathing were associated with a greater CSF tracer distribution into the cord via glymphatic flow, compared with the continuous positive pressures associated with mechanical ventilation [37]. Given that there is direct exposure of the epidural venous plexus to intrathoracic pressures [38], one would expect that negative intrathoracic pressures in spontaneous breathing would facilitate the drainage of the spinal cord venous blood and reduce vein size, thus accentuating glymphatic flow, whilst continuous positive-pressure ventilation would dilate the cord veins and reduce glymphatic flow. In humans, similar to the case in animal models, a marked dilatation of the perivascular spaces is seen adjacent to syrinxes post mortem [39].

Finally, an understanding of venous dilatation and its effect on glymphatic flow can explain some of the rarer but otherwise confusing causes of syringomyelia. Syringomyelia can develop from long-term lumboperitoneal shunting in those with Chiari 1 malformations [40] and can also occur with spontaneous intracranial hypotension developing from a lumbar nerve root diverticular sheath leak [41]. In both instances, a reduction in CSF pressure in the spinal canal compared to the cord draining venous pressure would dilate the cord veins and block the glymphatic flow.

### 2.3. The Complete Physiology of Syrinx Formation

Following the above explanation, a proposed complete physiology of syrinx formation will be explained using Figure 2.

The reduction in craniospinal compliance within the CSF space surrounding the spinal cord alters the impedance matching between the venule and the extradural veins dilating the venule due to an alteration in impedance pumping. The dilated vein narrows the perivenular space and increases the resistance to interstitial glymphatic fluid flow. The back-up of interstitial fluid increases the pressure in the interstitial space and there is a development of edema. The edema fluid begins to coalesce, forming small cystic spaces which eventually join up into a larger syrinx. The further back-up of the glymphatic fluid leads to arteriolar perivascular space dilatation.

### 2.4. Implications and Future Research Directions

The purpose of this proposed mechanism of syringomyelia is to highlight that treatments for this condition should be aimed at maximising craniospinal compliance. Similarly, as has been argued above, there is a similarity between the physiology of syringomyelia and that of hydrocephalus and multiple sclerosis. Therefore, the current findings would suggest that there are new areas to investigate in the cause of glymphatic dysfunction in the latter two diseases.

## 3. Conclusions

There is a correlation between hydrocephalus, multiple sclerosis and syringomyelia, with each having a reduction in craniospinal compliance. There is a dilatation of the outflow veins in hydrocephalus and multiple sclerosis with an associated reduction in glymphatic fluid flow. It is hypothesised that glymphatic flow abnormality is due to the shared venous and glymphatic outflow geometry. It is suggested that syringomyelia is analogous to the other two diseases.
